# O090: Impact on nurses of ebola outbreak

**DOI:** 10.1186/2047-2994-2-S1-O90

**Published:** 2013-06-20

**Authors:** D Pululu, S Mukendi, P Formenty, S Eremin, CL Pessoa-Silva

**Affiliations:** 1 Isiro General Hospital, Isiro, Congo, The Democratic Republic of the; 2 WHO, Geneva, Switzerland

## Introduction

On August 12, 2012, the DRC government declared *Bundibugyo ebolavirus* (BE) outbreak in Isiro city. As of October 15, 2012, 75 (33 confirmed, 17 probable and 25 suspected) cases, and 25 deaths had been reported among confirmed and probable cases.

## Objectives

To describe the occurrence of BE infection among nursess during the outbreak.

## Methods

A case series design was applied. Study period: 28 May to 15 October, 2012. Study population: 166 nurses in main HCFs in Isiro, HCF A (n=47, 70 beds), HCF B (n=32, 70 beds), HCF C (n=57, 160 beds), HCF D (n=30, 53 beds). Case definition established suspect, probable and confirmed (laboratory confirmed by RT-PCR or serology) cases. Active early case detection and infection prevention and control was promoted in all HCFs; an isolation unit established in HCF C on August 10, 2012 suspect cases were referred. Laboratory tests were performed in all suspect cases and HCWs who expressed interest. To assess potential risk factors, the HCFs in the Isiro urban-rural health zone were assessed for IPC practices and hand hygiene facilities, and a structured questionnaire applied to nurses who had been tested for BE infection to assess contact with BE cases, service and HCF where the nurse was assigned. Two-tailed exact test was used when appropriate.

## Results

26% of BE cases were HCWs, and nurses comprised 12/13 affected HCWs (7 confirmed, 5 probable cases). All cases among nurses occurred in HCF A (attack rate=15%) and HCF B (attack rate=12.5%) and 8 happened in the 1st month. 8/31 nurses tested were confirmed to be BE infected, and 2 had mild symptoms, not requiring hospitalization. 20/31 tested nurses acknowledged contact with a confirmed BE case, but only four had confirmed infection. No statistical significant difference was observed for service and history of contact. The only factor associated with BE infection among nurses was being a nurse in HCF A/B (p<0.001).

## Conclusion

This study confirms the HCWs and particularly nurses as at high risk for infection during Ebola epidemics. The occurrence of most infection in beginning of the outbreak when HCWs were not alert for the epidemic and IPC measures not in place highlights the importance of applying basic IPC precautions at all times.

## Disclosure of interest

None declared.

